# An immune genes signature for predicting mortality in sepsis patients

**DOI:** 10.3389/fimmu.2023.1000431

**Published:** 2023-02-13

**Authors:** Shirong Lin, Ping Li, Jibin Yang, Shiwen Liu, Shaofang Huang, Ziyan Huang, Congyang Zhou, Ying Liu

**Affiliations:** Department of Emergency, First Affiliated Hospital of Nanchang University, Nanchang, Jiangxi, China

**Keywords:** immune system, sepsis, gene signature, mortality, nomogram

## Abstract

A growing body of evidence indicates that the immune system plays a central role in sepsis. By analyzing immune genes, we sought to establish a robust gene signature and develop a nomogram that could predict mortality in patients with sepsis. Herein, data were extracted from the Gene Expression Omnibus and Biological Information Database of Sepsis (BIDOS) databases. We enrolled 479 participants with complete survival data using the GSE65682 dataset, and grouped them randomly into training (n = 240) and internal validation (n = 239) sets based on a 1:1 proportion. GSE95233 was set as the external validation dataset (n=51). We validated the expression and prognostic value of the immune genes using the BIDOS database. We established a prognostic immune genes signature (including ADRB2, CTSG, CX3CR1, CXCR6, IL4R, LTB, and TMSB10) *via* LASSO and Cox regression analyses in the training set. Based on the training and validation sets, the Receiver Operating Characteristic curves and Kaplan-Meier analysis revealed that the immune risk signature has good predictive power in predicting sepsis mortality risk. The external validation cases also showed that mortality rates in the high-risk group were higher than those in the low-risk group. Subsequently, a nomogram integrating the combined immune risk score and other clinical features was developed. Finally, a web-based calculator was built to facilitate a convenient clinical application of the nomogram. In summary, the signature based on the immune gene holds potential as a novel prognostic predictor for sepsis.

## 1 Introduction

Sepsis, which is defined as the systemic inflammatory response to infection, has been a leading cause of morbidity, mortality, and health system costs (1). Based on epidemiological studies, approximately 31.5 million cases of sepsis occur worldwide annually with a mortality rate of 16.8% (2). When sepsis becomes severe, it results in organ failure and a mortality rate of more than 20%, while when septic shock occurs, refractory hypotension develops and a mortality rate exceeding 40% occurs (3, 4). Sepsis treatments have advanced rapidly in the past few years, including antibiotic therapy, ventilator management, glucagon monitoring, and resuscitation strategies (5, 6). Nevertheless, there have been a few new effective therapies identified, and the mortality rate associated with sepsis remains high. Therefore, early identification of patients at high risk of death may be the key to preventing and treating sepsis patients and improving the survival rate of patients with sepsis (7). As sepsis is a complex and heterogeneous disease, it is often difficult for clinicians to accurately assess the risk of death (8). Therefore, it is vital to research novel biomarkers to better predict sepsis progression and improve sepsis patients’ prognosis.

A number of biomarkers are currently used as indicators of infection in critically ill patients, including the inflammatory marker C-reactive protein (CRP) and the bacteremia indicator procalcitonin (PCT); However, their diagnostic and prognostic abilities in the case of sepsis appear to be suboptimal (9, 10). It is currently unclear whether any biomarker can detect sepsis rapidly enough or identify high-risk patients in an acceptable manner, as sepsis has an extremely heterogeneous and complex pathophysiology. In recent years, with the accumulation of a large amount of “omics” data in public databases, gene expression signatures have been proven useful for predicting the mortality risk of different patients (11, 12). Theoretically, the heterogeneity of sepsis can be explained by the differential expression of thousands of genes in response to infectious agents (13). Hence, transcriptomics, as promising new biomarkers, can provide important predictive and prognostic information.

Increasing evidence currently supports the immune system’s core role in sepsis (14, 15). In sepsis, immune response activated by invading pathogens fails to return to homeostasis, thus ultimately leading to a pathological syndrome characterized by persistent excessive inflammation and immunosuppression (16). Immune-related genes (IRGs) are biologically important for the host’s response to pathogens and play key roles in immune responses (17). Therefore, researchers working on sepsis are becoming increasingly interested in IRGs and their prognostic value.

In this study, we investigated the difference in IRG expression in sepsis patients, and then developed a scoring model according to a multigene signature and other clinicopathological factors to improve prognosis prediction in sepsis patients, thereby facilitating clinical treatment. An estimation model was created using a nomogram and a web-based calculator, and its performance was evaluated according to its discrimination, calibration, and clinical significance.

## 2 Methods

### 2.1 Data collection

The gene expression arrays of human sepsis datasets were derived from inception to June 2022 based on the Gene Expression Omnibus (GEO, https://www.ncbi.nlm.nih.gov/geo/), which is a database of high-throughput gene expression data, hybridization arrays, microarrays, and chips is. A correction was made to all data before it was integrated. Additionally, the Biological Information Database of Sepsis (BIDOS, http://www.swmubidos.com/) was applied to validate the expression and prognostic value of IRGs in sepsis. In total, 2498 immune-related genes were downloaded from ImmPort (https://immport.niaid.nih.gov), which includes a comprehensive list (18).

### 2.2 Differential expression analysis

The probes with missing gene symbols and missing values were removed. In order to standardize the data, we used the robust multiarray (RMA) approach. Differentially expressed genes were determined *via* the Limma package of R language (19), with |log 2 (fold change [FC]) | > 0.5 and p< 0.05 as the significant thresholds.

### 2.3 Functional enrichment analysis

GO analysis, which incorporated biological processes, molecular functions, and cellular components, and Kyoto Encyclopedia of Genes and Genomes (KEGG) pathway enrichment analyses were performed by using the clusterProfiler package in R language (20), set the significant threshold to p< 0.05.

### 2.4 Construction of the immune signature

Patients with follow-up duration and status in GSE65682 were randomly divided into two groups: training and internal validation, and other GEO datasets were set as external validation sets. To screen for immune genes associated with survival, a univariate Cox regression analysis was performed using the “survival” package. By using the R packages “glmnet” and “survival,” the LASSO regression analysis was carried out to screen potential genes based on variable screening and complexity adjustment. As a final step, a multivariate Cox regression analysis was conducted to confirm the identity of highly correlated genes and construct an immune gene signature based on the following model:


$$\text{Risk score}=\sum_{\text{i}=0}^{N}\left(\beta_{i}\times Exp_{i}\right)$$

where N indicates the number of IRGs included, and Expi indicates the level of the mRNA for each of these genes. βi represents the regression coefficient obtained using the Cox regression method.

The median risk score was used as a cutoff for dividing patients into high- and low-risk groups, and receiver operating characteristic (ROC) curves were produced using the “survivalROC” package in R. Assessment of the immunogene signature’s predictive potential was conducted using AUC values.

### 2.5 Immune cell infiltration

The immune cell infiltration in the high- and low-risk groups was studied using the CIBERSORT algorithm (HTTPS://cibersort.stanford.edu/) (21). A landscape map was used to illustrate differences between high-risk and low-risk groups in the relative proportions of 22 types of immune cells.

### 2.6 Statistical analysis

By using the median risk score in each data set as a cutoff, Kaplan-Meier curves were plotted to compare the survival risks between high-risk and low-risk individuals. To determine whether multi-mRNA signatures and clinicopathologic characteristics were independent factors, both univariate and multivariate Cox regressions were applied. Significance was defined as P< 0.05. R software version 4.0.5 was used for all analyses except those requiring special parameters.

## 3 Results

### 3.1 Identification of differentially expressed genes

**Figure 1:**
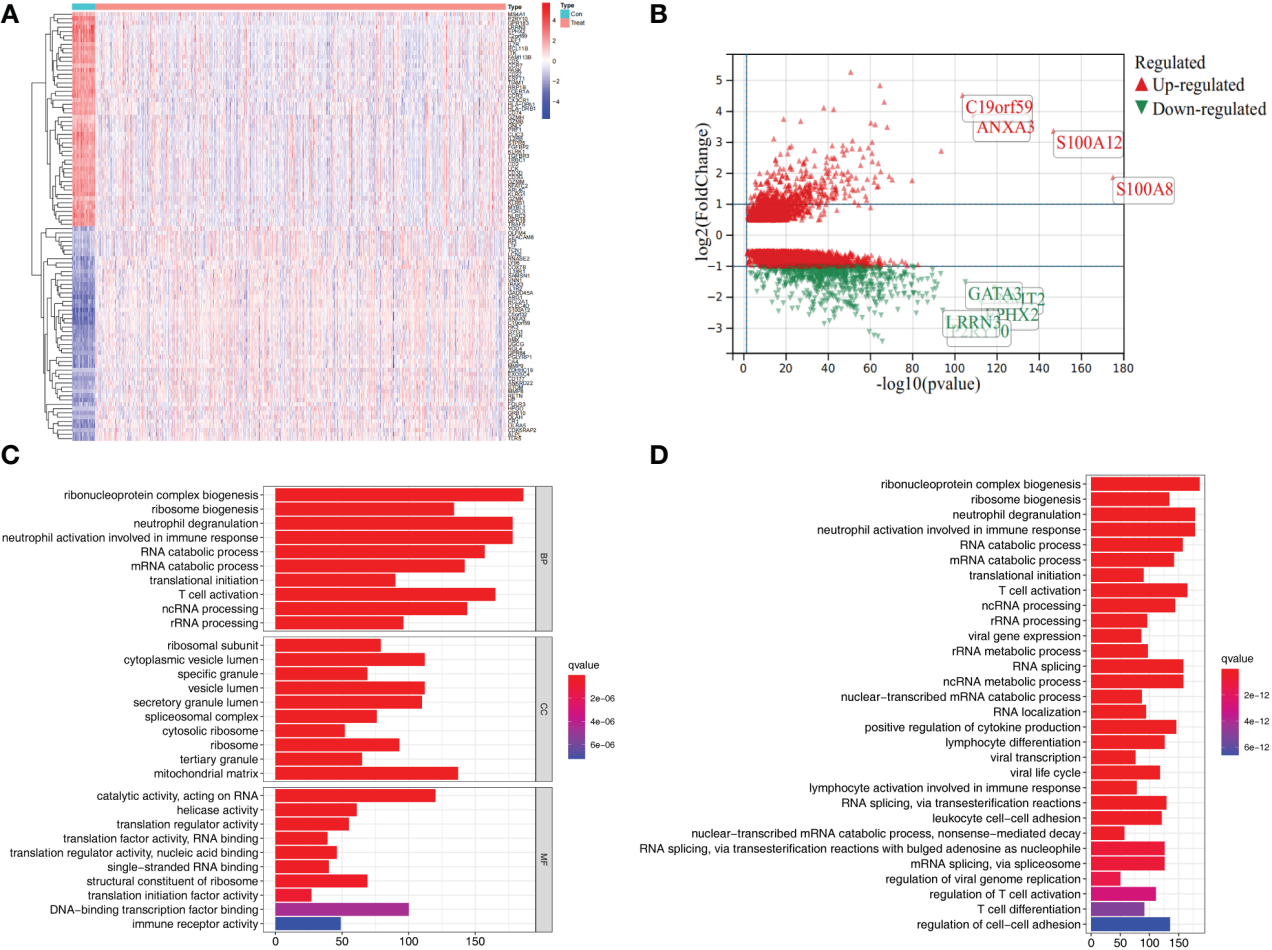
Differential gene expression and functional enrichment analysis in the GSE65682 dataset. **(A)** Heat map showing top 50 differential expression genes in control and sepsis. **(B)** Volcano plots showing differential expression genes (logFC>0.5). **(C)** GO enrichment results of differential expression genes. **(D)** KEGG pathway enrichment analysis of differential expression genes.

To identify the differentially expressed genes, we detected the differentially expressed genes between control and sepsis samples in GSE65682. We initially identified 3,648 differentially expressed genes, of which 1,187 were upregulated and 2,461 were downregulated (**Figures 1A, B**). GO, and KEGG functional enrichment analysis revealed that this set of genes was mainly enriched in immune/inflammation-related functions (**Figures 1C, D**).

### 3.2 Construction of immune signature

To screen out differentially expressed immune genes, 283 genes were selected by taking the intersection of the 3,648 differentially expressed Genes and the 2,498 immune genes (**Figure S1**). Next, univariate Cox regression analysis was used to screen the immune genes with prognostic values based on the training set. Eventually, 28 survival-related IRGs were identified (**Table S1**). Subsequently, the 28 IRGs were used in Lasso-Cox proportional hazards regression and ten-fold cross-validation analyses designed to construct the best gene signature; eventually, 14 IRGs were identified for downstream analysis (**Figure S1**). Furthermore, multivariate analysis was performed based on the Lasso results using the Cox Proportional hazards model, and 7 immune genes were identified to construct the prognostic model (**Figure S1**). In addition, we also explored the correlations between the 7 genes. The heatmap of gene expression correlation is illustrated in **Figure S1**.

### 3.3 Diagnostic value of IRG

**Figure 2:**
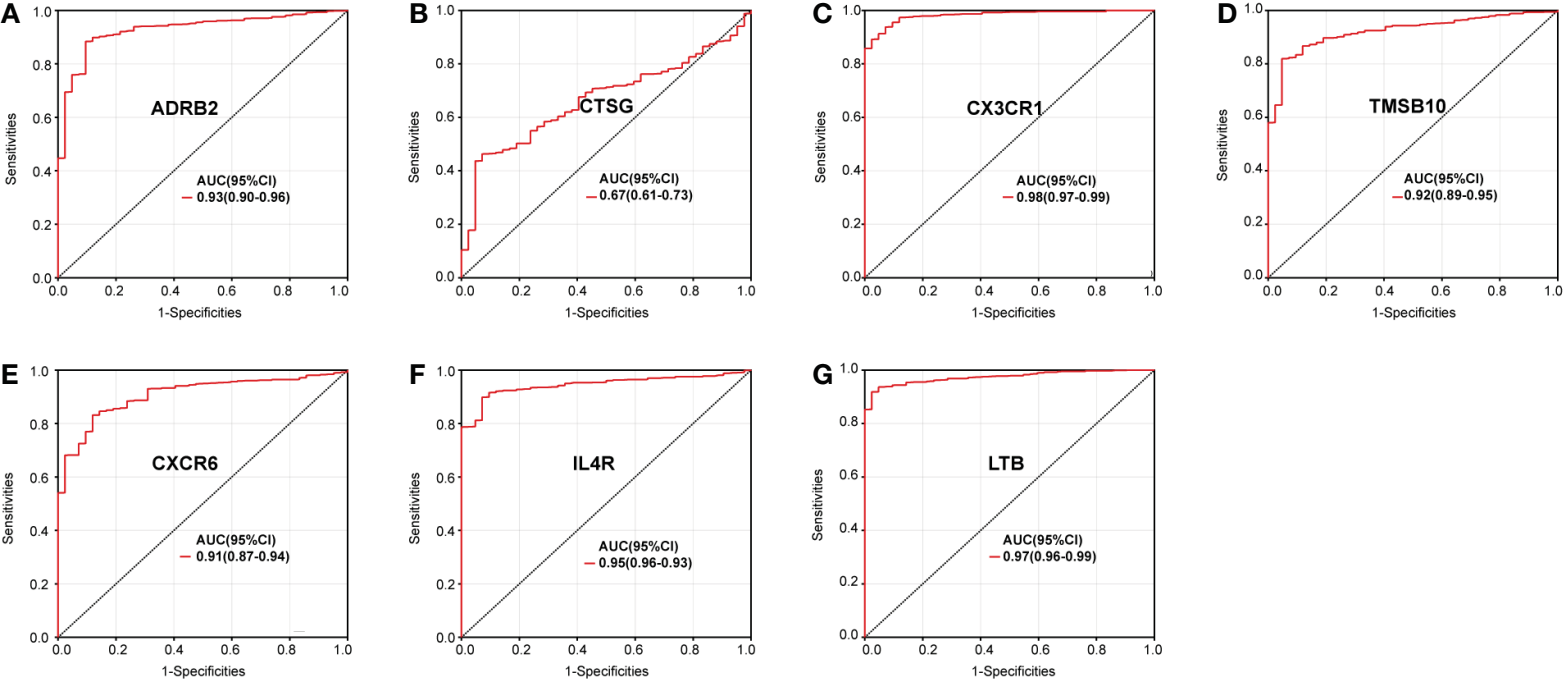
Diagnostic value of the immune genes for sepsis assessed *via* ROC curve analysis: **(A)** ADRB2, **(B)** CTSG, **(C)** CX3CR1, **(D)** CXCR6, **(E)** IL4R, **(F)** LTB, and **(G)** TMSB10.

We further investigated the diagnostic effectiveness of the seven identified IRGs (ADRB2, CTSG, CX3CR1, CXCR6, IL4R, LTB, and TMSB10). As displayed in **Figures 2A–G**, the diagnostic ability of each IRG to distinguish sepsis from the control samples shows a superior diagnostic efficiency. ROC curve analysis revealed that the area under the curve (AUC) was 0.93 (95% confidence interval [CI], 0.90–0.96) for ADRB2, AUC was 0.67 (95% CI, 0.61–0.73) for CTSG, AUC was 0.98 (95% CI, 0.97–0.99) for CX3CR1, AUC was 0.92 (95% CI, 0.89–0.95) for TMSB10, AUC was 0.91 (95% CI, 0.87–0.94) for CXCR6, AUC was 0.95 (95% CI, 0.93–0.96) for IL4R, and AUC was 0.97 (95% CI, 0.96–0.99) for LTB.

### 3.4 Validation of the expression of IRGs

**Figure 3:**
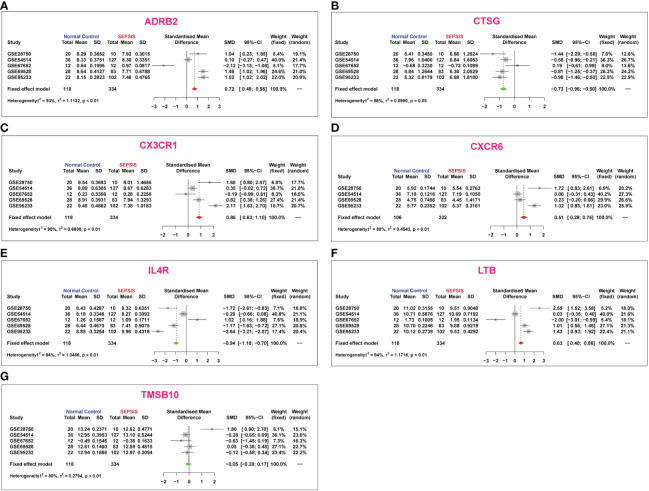
Meta-analysis of immune gene expression in sepsis based on existing microarray data sets: **(A)** ADRB2, **(B)** CTSG, **(C)** CX3CR1, **(D)** CXCR6, **(E)** IL4R, **(F)** LTB, and **(G)** TMSB10.

A meta-analysis of IRG levels was performed to validate the gene expression level between control and sepsis. We screened the research data set of patients with sepsis in the GEO database. Eventually, the following data sets: GSE28750, GSE6535, GSE12624, GSE54514, GSE63042, GSE67652, GSE69528, GSE74224, and GSE95233 were included. After pooling the mRNA level of IRGs from the different datasets, we found nearly the same trend in the validation set (**Figures 3A–G**).

### 3.5 Validation of the prognostic role of immune genes

**Figure 4:**
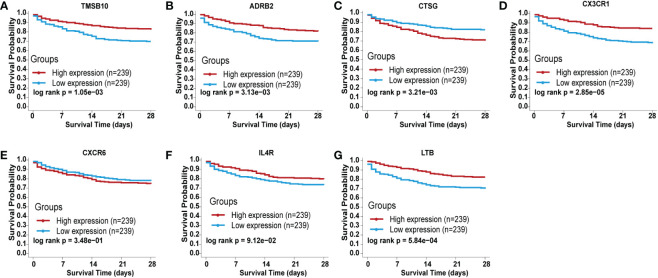
Validation of the independent prognostic efficiency for individual mRNA in the 7-immune gene signature based on the BIDOS database. **(A)** ADRB2, **(B)** CTSG, **(C)** CX3CR1, **(D)** CXCR6, **(E)** IL4R, **(F)** LTB, and **(G)** TMSB10.

To validate the prognostic role of each identified IRGs in sepsis, we searched the BIDOS database, which included gene expression data and survival data of sepsis obtained from the GEO database. The K-M analysis results showed that most of the IRGs have a significant prognostic value (**Figures 4A–G**).

### 3.6 Prognostic value of the IRG signature

**Figure 5:**
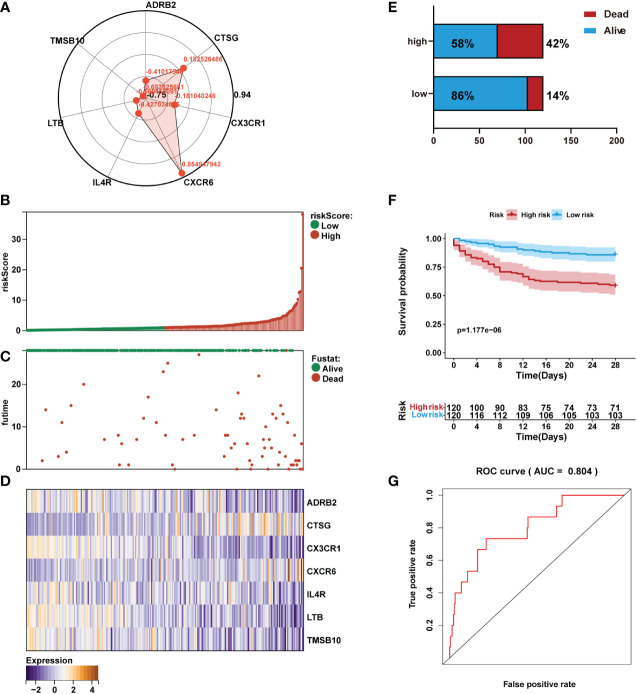
Prediction performance assessment of the prognostic signature in the training set (n=240). **(A)** Coefficient distribution of the gene signature. **(B)** Bar plot of a risk score for every patient and ordered by the value of risk score. **(C)** Distribution of vital survival status and risk scores of the immune signature. **(D)** Heatmap of the mRNA expression levels of the seven signature-comprising immune genes. **(E)** Comparison of survival risk between two groups. **(F)** Kaplan-Meier survival analysis of the gene signature in the training set. **(G)** ROC curves of the seven-mRNA signature in sepsis.

According to Cox multivariate analysis, the Cox coefficients of the 7 IRGs were obtained (**Figure 5A**). The prognostic risk score according to the expression of the 7 IRGs was determined as follows: Risk score = ADRB2 × (-0.4102) + CTSG × (0.1825) + CX3CR1 × (-0.1810) + CXCR6 × (0.8549) + IL4R × (-0.4270) + LTB × (-0.5605) + TMSB10 × (-0.6836). The gene expression levels in the formula of the risk score were normalized microarray data.

The risk score for each sepsis patient in the training set was calculated. Patients were separated into high- and low-risk cohorts based on the median risk score. We plotted the patient’s risk score (green for low-risk values, red for high-risk values) and survival state chart (green for alive, red for death) (**Figures 5B, C**). Differences in expression of the seven IRGs between low- and high-risk are shown in **Figure 5D**. The results showed sepsis exhibited a greater mortality risk with an increasing risk score (**Figure 5E**).

Kaplan-Meier analysis revealed that in the training set, patients in the high-risk group had shorter overall survival (OS) than those in the low-risk group (**Figure 5F**). Receiver operating characteristic (ROC) analysis demonstrated that the area under the curve (AUC) value of the prognostic immune signature was 0.804 (**Figure 5G**).

### 3.7 Validation of the IRG signature

**Figure 6:**
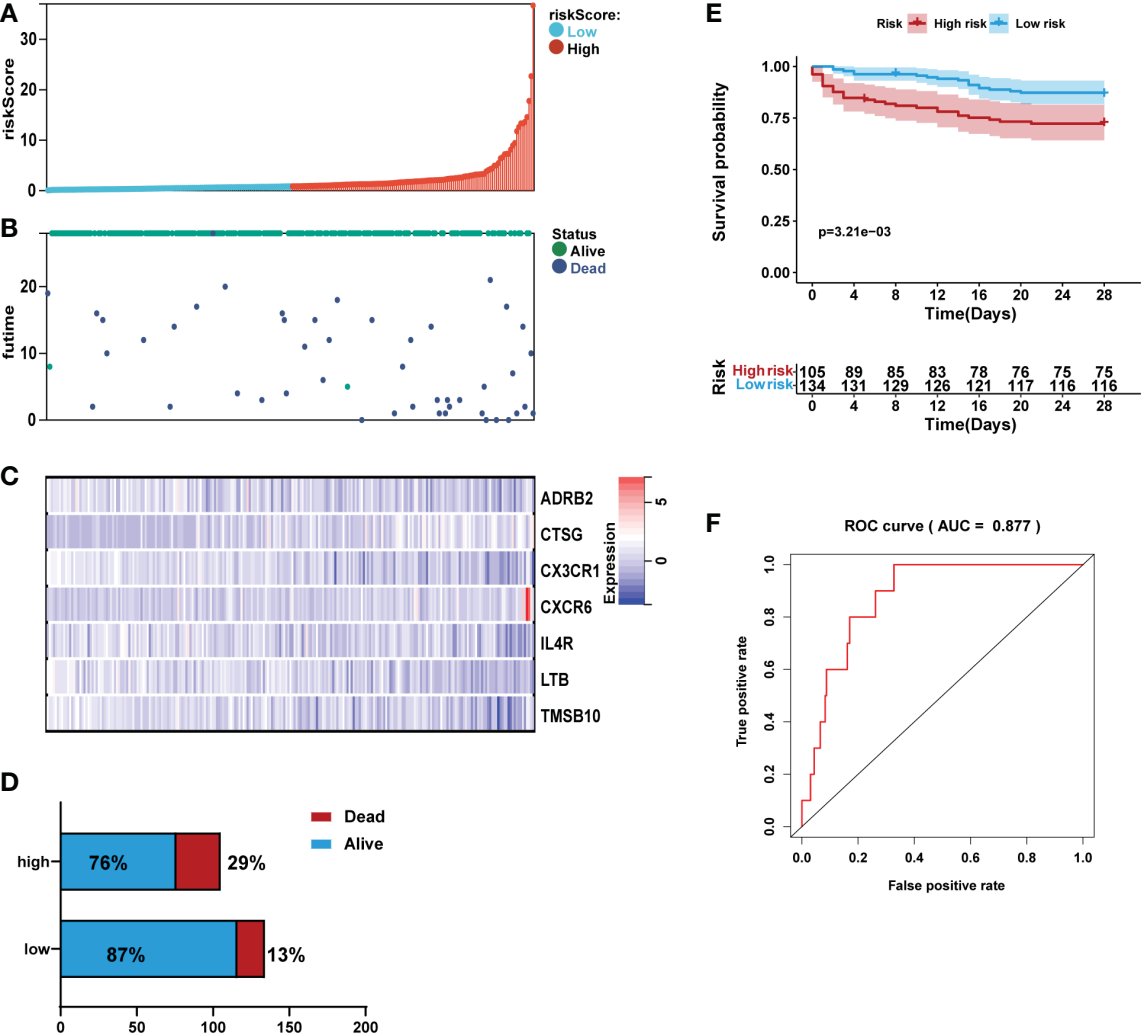
Validation of the prediction performance of the prognostic signature in the validation set (n=239). **(A)** Bar plot of a risk score for every patient and ordered by the value of risk score. **(B)** Distribution of vital survival status and risk scores of the immune signature. **(C)** Heatmap of the mRNA expression levels of the seven signature-comprising immune genes. **(D)** Comparison of survival risk between two groups. **(E)** Kaplan-Meier survival analysis of the gene signature in the validation set. **(F)** ROC curves of the seven-mRNA signature in sepsis.

The risk score for each sepsis patient in the validation set was calculated to validate the IRG signature. Samples were separated into high- and low-risk cohorts based on the median risk score. We plotted the patient’s risk score (green for low-risk values, red for high-risk values) and survival state chart (green for alive, red for death) in **Figures 6A, B**. Differences in expression of the seven IRGs between low- and high-risk are shown in **Figure 6C**. The results indicated a higher mortality risk with an increasing risk score (**Figure 6D**).

Kaplan-Meier analysis revealed that in the validation set, patients in the high-risk group had high mortality than those in the low-risk group (**Figure 6E**). ROC analysis demonstrated that the area AUC value of the prognostic signature was 0.877 (**Figure 6F**).

**Figure 7:**
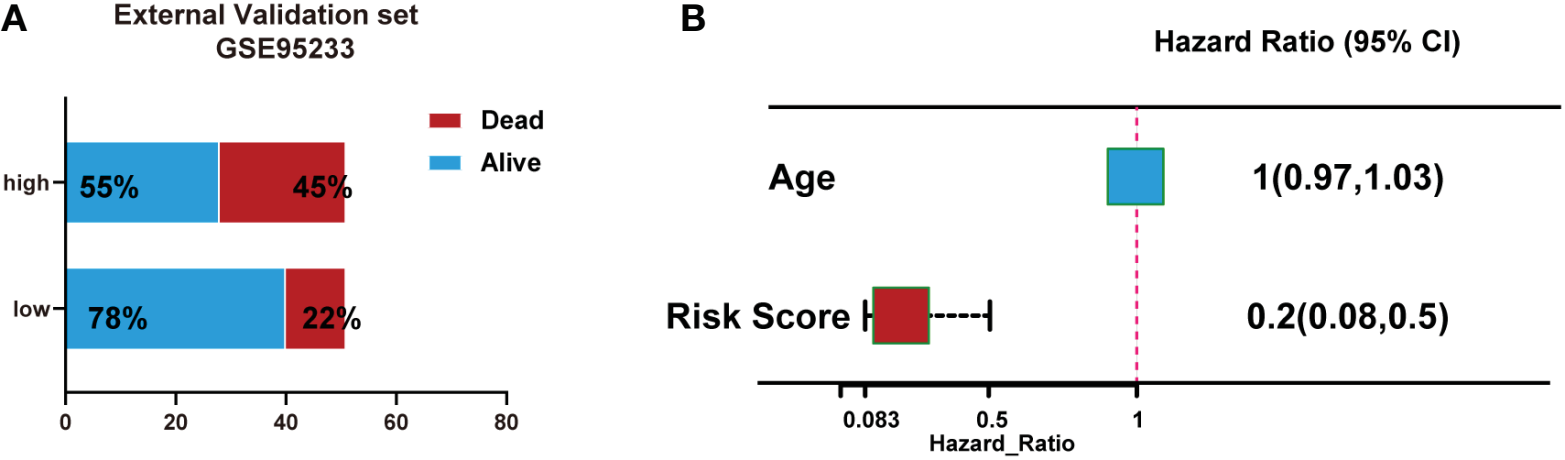
Validation of the signature using an external validation dataset (GSE95233). **(A)** Number of patient deaths increased with rising risk scores. **(B)** Multivariate logistic regression analysis showed that risk score was independently associated with survival.

We also validated the signature by an external validation data set (GSE95233). The results showed a higher portion of dead patients with increasing risk scores (**Figure 7A**). Moreover, Multivariate logistic regression analysis showed that risk score was independently associated with survival (**Figure 7B**).

### 3.8 Immune cell infiltration estimation

To further study immune characteristics in sepsis with a different immune risk score. We investigated the infiltrating immune cells by using the CIBERSORT algorithm. The percentage of immune cells that infiltrated the tumor is shown in **Figure S2**. Based on immune infiltration levels among the different immune infiltrating cells, we compared the high-risk group with the low-risk group. The results showed that the high-risk group had high proportions of plasma cells and macrophages M1. And the low-risk group had high proportions of T cells CD8, NK cells resting, and macrophages M0 (**Figure S2**).

### 3.9 Development of prediction nomogram and web-based calculator

**Figure 8:**
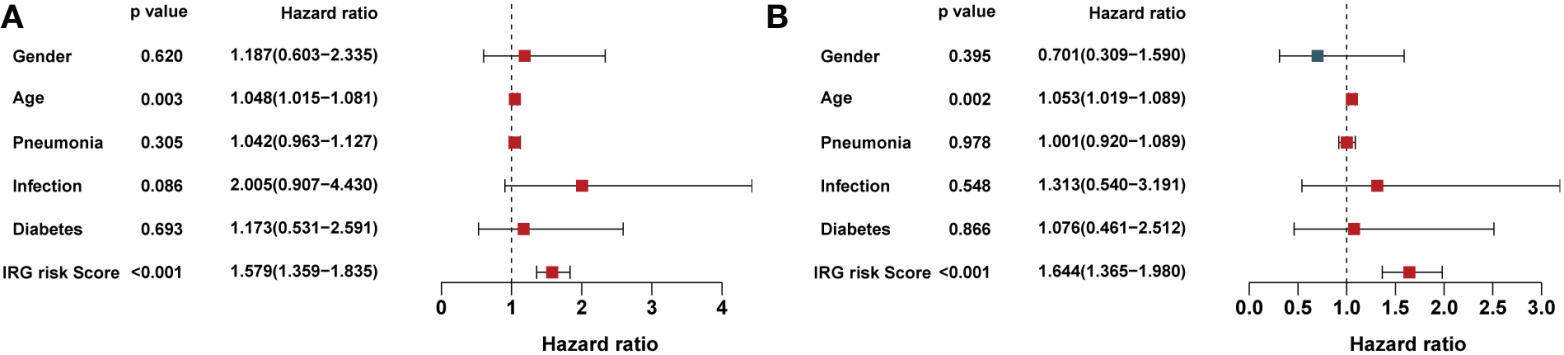
Univariate and multivariate analysis of the influence of specific clinical characteristics on the outcome of sepsis: **(A)** Univariate analysis and **(B)** multivariate analysis.

**Figure 9:**
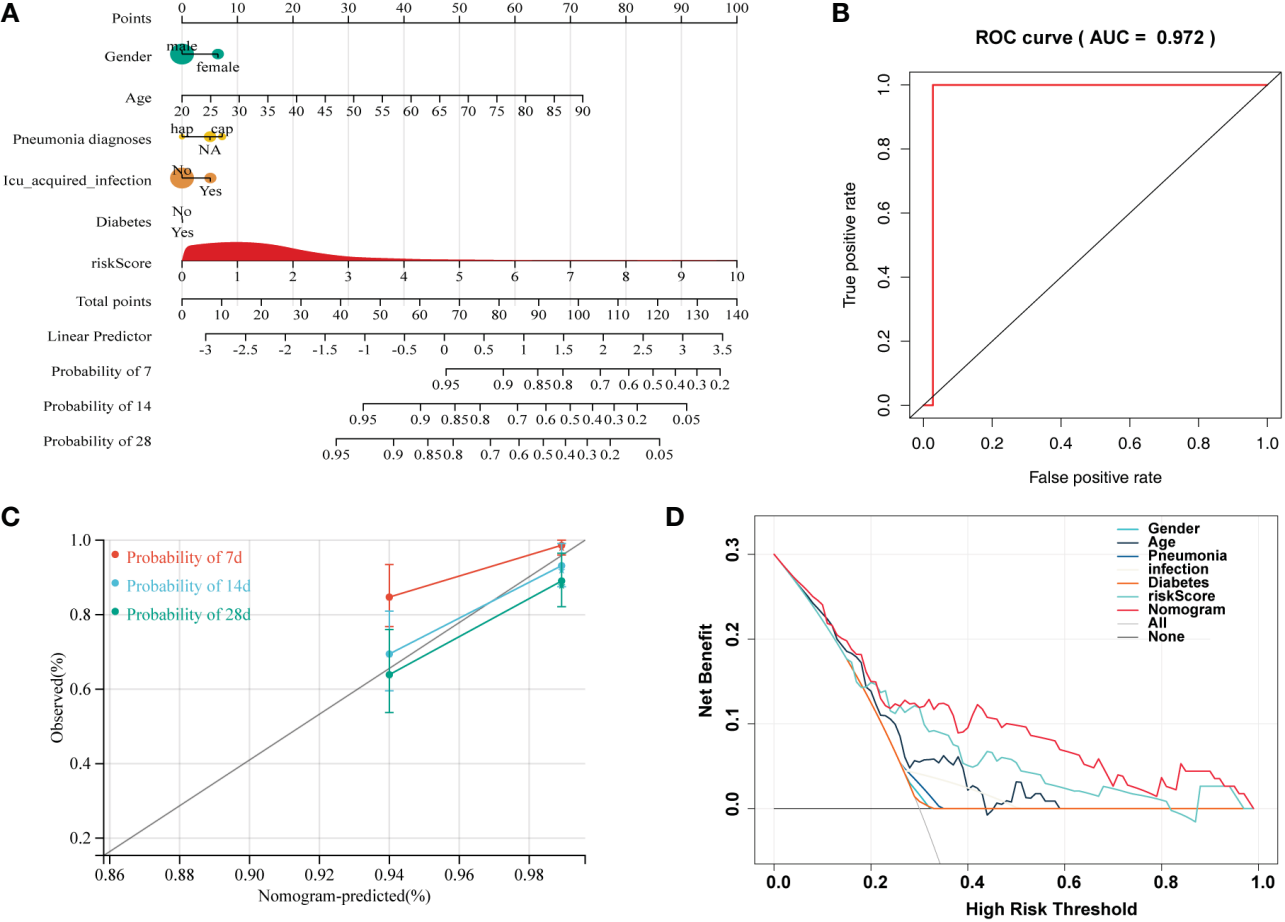
A nomogram developed for predicting sepsis mortality. **(A)** Nomogram for predicting 7-, 14-, and 28- days survival based on the immune gene signature and clinical features. **(B)** ROC curve for survival prediction of the nomogram. **(C)** Calibration plot of the nomogram for the prediction of survival. **(D)** DCA of the nomogram for the prediction of survival.

Independent prognostic factors were identified by Cox proportional hazards regressions. The results showed this immune risk signature was independent of prognostic factors (**Figures 8A, B**). A nomogram was established to accurately predict a certain clinical outcome by integrating age, gender, ICU-acquired infection, diabetes, and the risk signature using a Cox model (**Figure 9A**). In the training set, the AUCs of the nomogram were 0.972 (**Figure 9B**). The calibration plots based on the training set showed good agreement between predictions and observations (**Figure 9C**). Further, a decision curve analysis (DCA) was conducted and showed that nomogram was clinically useful (**Figure 9D**).

Moreover, we built an easy-to-use web-based resource (https://emergency.shinyapps.io/sepsis/) for clinical use and visualization of the prediction model to estimate the mortality risk of sepsis based on the nomogram (**Figure S3**). The estimated probabilities of disease progression may be derived by drawing a perpendicular line from the total point axis to the outcome axis.

## 4 Discussion

ICU deaths are mainly caused by sepsis, including septic shock, which has a poor patient prognosis (22). There are many therapies for the treatment of sepsis. However, no specific medicine has been formulated thus far. When managing sepsis, it is critical to diagnose sepsis early and recognize patients with high mortality risk (23). In this study, we established robust prognostic seven-immune genes (including ADRB2, CTSG, CX3CR1, CXCR6, IL4R, LTB, and TMSB10). In addition, the IRGs displayed excellent diagnostic capability for sepsis.

Studies in the past have attempted to develop models of prognosis in sepsis patients based on new biomarkers and clinical features (24–27). However, due to the reliability, limited results have been applied in clinical practice. In this study, our signature showed a high predictive ability for sepsis. The AUC obtained by our signature ranged from 0.804 to 0.877, with an average AUC value of 0.840. More importantly, we built a more reliable nomogram model by incorporating clinical risk factors and risk scores, with the AUC reaching 0.972 and developed an easy-to-use calculator which will allow the public to freely predict local cases and test the model’s adaptability.

Most IRGs included in our signature were closely related to inflammation and immune response. ADRB2 belonged to superfamily A of seven transmembrane G protein-coupled receptors (GPCRs) activated by epinephrine (Epi) or norepinephrine (NE) (28). Immune cells express ADRB2, enabling the sympathetic nervous system to control immune function directly (29). Previous studies indicated that ADRB2 controls inflammation by driving rapid IL-10 secretion (30). CTSG is a recognized neutrophil protease released by activated neutrophils to clear pathogens and regulate inflammation by modifying chemokines, cytokines, and cell surface receptors (31). We found the upregulated CTSG expression in sepsis, which might be a regulator of inflammation response during sepsis. CX3CR1 is a chemokine receptor that binds to the proinflammatory chemokine fractalkine (FKN or CX3CL1) (32). A previous study demonstrated that the internalization of CX3CR1 is closely associated with immunoparalysis in the late phase of sepsis (33). CXCR6 is the receptor found on cells at sites of inflammation. There were limited studies that focused on the role of CXCR6 plays in sepsis. In our study, it was found that CXCR6 was downregulated when compared to the control. IL-4R, a specific receptor of inflammatory factors IL-4, transmits signals into the cellular nucleus and exerts biological functions. IL4/IL4 receptor (IL4R) interaction has well-defined roles in the immune system. In our research, the result showed the upregulated IL4R expression in sepsis. However, the specific role and mechanism of IL4R in sepsis requires further elucidation. LTB is a lipid mediator produced quickly (seconds to minutes) by phagocytes and induces chemotaxis, and blocking LTB4 actions could be a promising therapeutic strategy to prevent inflammasome-mediated diseases (34). TMSB10 was originally identified in the thymus, which plays a key role in the immune system (35). Most of the previous research on TMSB10 has focused on the role of cancer immune microenvironmental, and the critical role in sepsis still needs further study.

The IRG plays an important role in regulating immune cell function and response (17, 36). Here, we evaluated the differences in the proportions of 22 immune cell types between low- and high-risk sepsis groups. Our results showed that the high-risk group had high proportions of plasma cells and macrophages M1, and the low-risk group had high proportions of T cells CD8, NK cells resting, and macrophages M0. These results implied that high portions of plasma cells and macrophage M1 are associated with poor prognosis.

Nomogram is a simple tool that can estimate risk by creating a visible picture; it is frequently used in clinical practice (37, 38). It incorporates several key features and is a simple and easy-to-use tool that clinicians may use to diagnose and estimate various patient groups’ prognoses. In this study, we created a nomogram based on the immune risk score and other clinical features to estimate the survival of patients with sepsis. In addition to the classic nomogram, we created a dynamic nomogram that could estimate patient prognosis using a simple web page operation. Unlike previous nomograms that calculated an estimate, the dynamic nomogram may deliver exact results.

However, several limitations need to be mentioned. First, all the cases used in this research were downloaded from an open accessed dataset, but none of our data were used for external verification. Second, *in vitro* or *in vivo* studies need to be performed to investigate the critical mechanisms associated with the prognostic significance of the identified immune genes in sepsis.

In summary, our study identified and validated an IRG signature that could independently predict the mortality risk of sepsis patients. A prognostic nomogram was constructed by integrating the immune risk score and other clinical features, which performed well in predicting the survival of sepsis patients. Therefore, we fabricated a clinically useful tool for improving the prognostic management of sepsis.

## Data availability statement

The original contributions presented in the study are included in the article/**Supplementary Material**. Further inquiries can be directed to the corresponding author.

## Author contributions

SRL and YL involved in study concept and design. PL, JY, SWL, and SH involved in acquisition of data. ZH and CZ involved in analysis and interpretation of data. SRL and YL drafted the manuscript. SRL and YL involved in critical revision of the manuscript for intellectual content. All authors contributed to the article and approved the submitted version.
