# Supplementary material for: An immune genes signature for predicting mortality in sepsis patients
Source: Front Immunol. 2023 Feb 13;14:1000431. doi: 10.3389/fimmu.2023.1000431 (PMC9968838; doi:10.3389/fimmu.2023.1000431)
Supplement: Supplementary file 1 [file DataSheet_1.pdf]

Table S1. Survival related immune gene based on univariate Cox analysis.

Symbol	HR	95% CI		pvalue
ADRB2	0.6345	0.4558-	0.8833	0.0070
ARRB1	0.5027	0.3121-	0.8096	0.0047
AZU1	1.2788	1.0791-	1.5155	0.0045
CD74	0.6751	0.5190-	0.8781	0.0034
CD81	0.6540	0.4898-	0.8733	0.0040
CTSG	1.1755	1.0504-	1.3156	0.0049
CX3CR1	0.6985	0.5854-	0.8334	0.0001
CXCR6	1.7839	1.1518-	2.7627	0.0095
DEFA4	1.1517	1.0371-	1.2789	0.0082
ELANE	1.2014	1.0648-	1.3556	0.0029
HCK	0.5860	0.4147-	0.8280	0.0024
HLA-DPB1	0.6899	0.5234-	0.9093	0.0084
HLA-DRA	0.7479	0.6010-	0.9307	0.0092
HLA-DRB1	0.7498	0.6160-	0.9127	0.0041
HLA-F	0.5763	0.3880-	0.8560	0.0063
IL16	0.4964	0.2971-	0.8296	0.0075
IL4R	0.6149	0.4325-	0.8742	0.0067
ISG20L2	0.4966	0.3169-	0.7780	0.0022
ITGAL	0.3927	0.2403-	0.6416	0.0002
LTB	0.5276	0.3889-	0.7156	0.0000
MPO	1.2524	1.0842-	1.4468	0.0022
OPRL1	0.5623	0.3937-	0.8030	0.0015
PAK1	0.5558	0.4038-	0.7650	0.0003
PROK2	0.7488	0.6096-	0.9198	0.0058
PTX3	1.3293	1.0900-	1.6212	0.0049
RNASE3	1.2572	1.0691-	1.4784	0.0056
TFRC	1.3655	1.1316-	1.6478	0.0012
TMSB10	0.4222	0.2445-	0.7290	0.0020
TNFSF12	0.3260	0.1779-	0.5976	0.0003

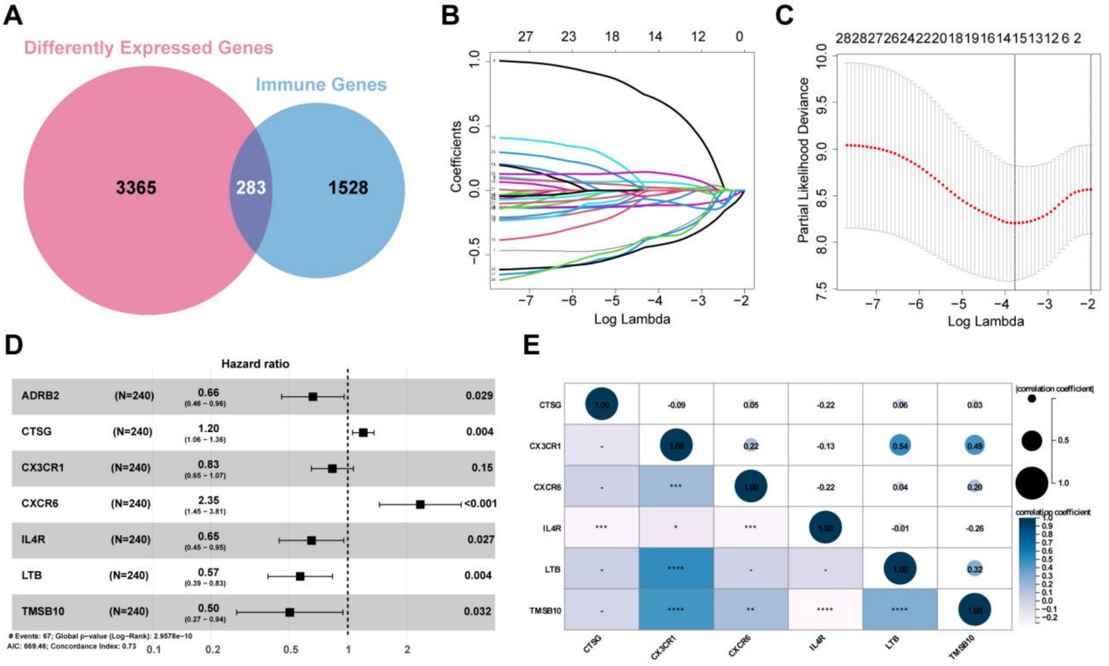

Figure S1: Construction of Prognostic immune Signature in the training set (n=240). (A) Venn diagram of differentially expressed immune genes. (B) Identification of 14 prognostic immune genes using LASSO regression analysis based on the Univariate Cox regression results. (C) Cross-validation of gene selection using 1-SE criteria in the LASSO regression analysis. (D) Forest plot of multivariate Cox regression analysis of the genes in the immune signature. (E) Heatmap showing the correlation of the immune genes.

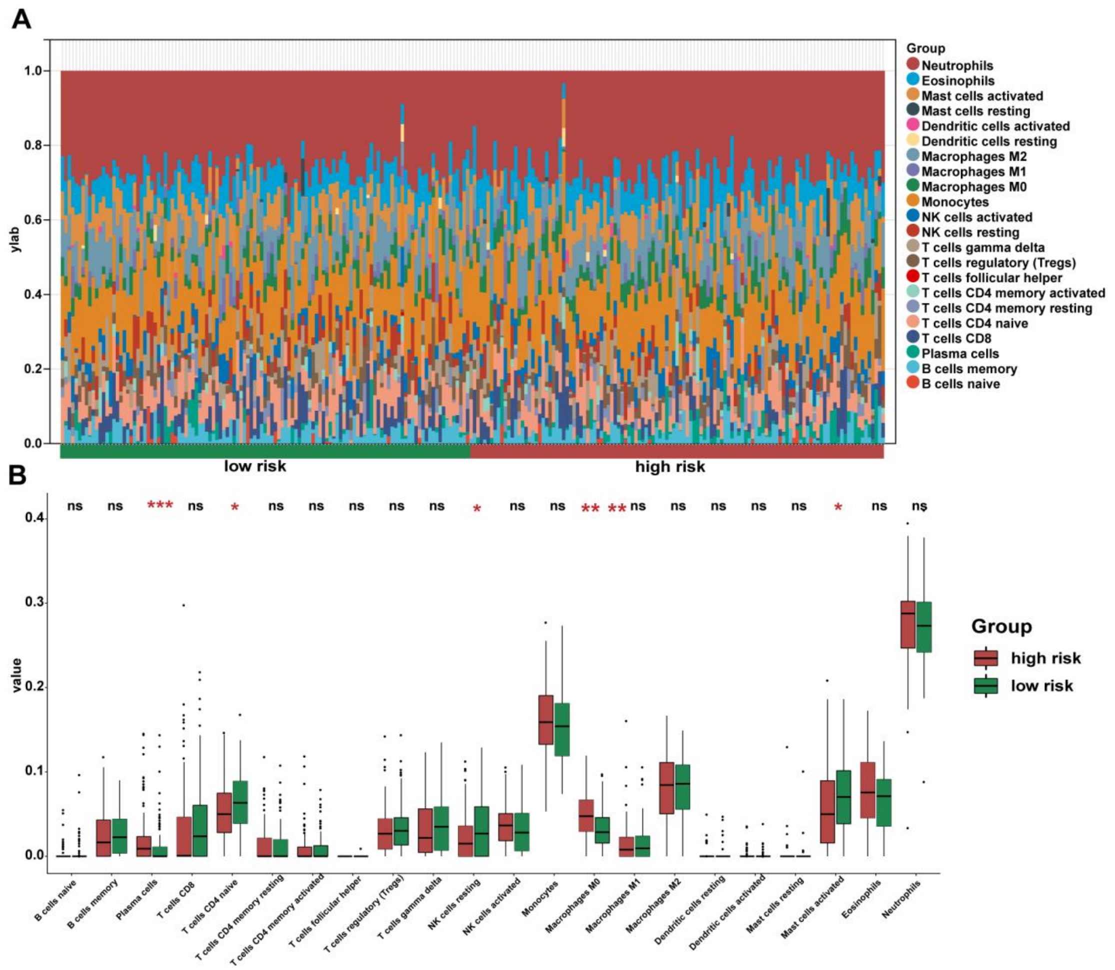

Figure S2: Differences in the immune cell infiltration between low- and high-risk groups of sepsis. (A) Relative proportions of immune cells between different groups. (B) Vioplot visualization of significantly different proportions of immune cells between different groups (*<0.05, **<0.01, and ***<0.001).

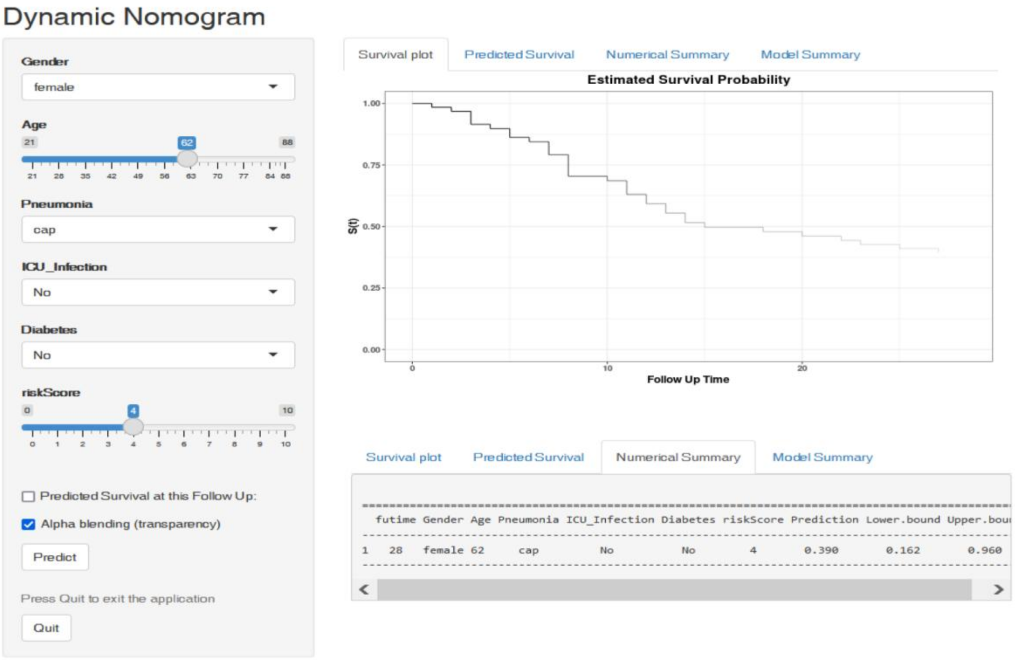

Figure S3: Web-based dynamic nomogram for estimating the probability of mortality . By entering the specifics of the sepsis into the web-based program, we determined the participant's survival probability (Entering Interface: This interface allows you to enter participant-specific information. Survival probability, as depicted graphically in part, (https://emergency.shinyapps.io/sepsis/).
